# EMOTIONAL CLOSENESS, SOCIAL NETWORK, NEGATIVE AND POSITIVE AFFECTS, AND AGITATION IN OLDER ADULTS WITH DEMENTIA

**DOI:** 10.1093/geroni/igad104.2723

**Published:** 2023-12-21

**Authors:** Mohammad Rababa

**Affiliations:** Jordan University Of Science And Technology, Irbid, Irbid, Jordan

## Abstract

**Background:**

If given a choice, people with dementia (PWD) would prefer to live in their homes instead of nursing homes. Living in older adults’ own houses would positively impact their quality of life and be associated with more privacy, security, and social support. In addition, living in a nursing home is not culturally and religiously accepted, especially in a religious and conservative country like Jordan. However, with increased financial obligations and hardships in Jordan, women are forced to work outsides their houses. Therefore, PWD stay alone in their homes without care or attention. This neglect would put PWD at significant risk for adverse physical and psychosocial health consequences. Method: This descriptive cross-sectional study was conducted on a convenience sample of 102 Jordanian PWD (51 Community-dwelling older adults and 51 NH residents) to examine the association of their emotional closeness and social networks with agitation and positive and negative affects. Result: The finding of this study based on an independent samples t-test showed that NH residents were significantly more likely to have physically non-aggressive behaviors than community-dwelling older adults. The study showed that the PWD’s sex (male), higher educational levels, and patients with a family member in the healthcare field are associated significantly with their mean positive affects score.

**Conclusion:**

Future research would consider exploring potential factors associated with agitation and affect in PWD. Experimental studies that intend to increase the levels of social support and emotional closeness, thus alleviating negative affect and levels of agitation, are recommended in the future.

